# Role of exosomes in modulating non-small cell lung cancer radiosensitivity

**DOI:** 10.3389/fphar.2024.1471476

**Published:** 2024-12-16

**Authors:** Jincheng Fang, Xinrui Rao, Changjian Wang, Yangchenxi Wang, Chuangyan Wu, Rui Zhou

**Affiliations:** ^1^ Department of Thoracic Surgery, Union Hospital, Tongji Medical College, Huazhong University of Science and Technology, Wuhan, China; ^2^ Cancer Center, Union Hospital, Tongji Medical College, Huazhong University of Science, Wuhan, China; ^3^ Institute of Radiation Oncology, Union Hospital, Tongji Medical College, Huazhong University of Science, Wuhan, China; ^4^ Hubei Key Laboratory of Precision Radiation Oncology, Wuhan, China

**Keywords:** non-small cell lung cancer, exosomes, radiotherapy, radioresistance, cancer

## Abstract

Non-small cell lung cancer (NSCLC) constitutes a significant proportion of lung cancer cases, and despite advancements in treatment modalities, radiotherapy resistance remains a substantial hurdle in effective cancer management. Exosomes, which are small vesicles secreted by cells, have emerged as pivotal players in intercellular communication and influence various biological processes, including cancer progression and the response to therapy. This review discusses the intricate role of exosomes in the modulation of NSCLC radiosensitivity. The paper focuses on NSCLC and highlights how tumor-derived exosomes contribute to radioresistance by enhancing DNA repair, modulating immune responses, and altering the tumor microenvironment. We further explore the potential of mesenchymal stem cell-derived exosomes to overcome radiotherapy resistance and their potential as biomarkers for predicting therapeutic outcomes. Understanding the mechanisms by which exosomes affect radiotherapy can provide new avenues for enhancing treatment efficacy and improving the survival rates of patients with NSCLC.

## 1 Introduction

Lung cancer remains the leading cause of cancer-related mortality worldwide, highlighting its critical impact on public health. The burden associated with this disease is substantial, with non-small cell lung cancer (NSCLC) accounting for approximately 85% of all lung cancer cases and representing the predominant subtype (Siegel et al., 2023). The current therapeutic strategies for NSCLC include a multidisciplinary approach that encompasses surgery, chemotherapy, radiotherapy, immunotherapy, and targeted therapies (Memon et al., 2024; Vinod and Hau, 2020; Herbst et al., 2018). Radiotherapy significantly improves local control and overall survival rates in patients and plays a pivotal role in the management of NSCLC (Aupérin et al., 2010). Especially, in the era of immunotherapy, combining radiotherapy with immunotherapeutic agents not only significantly enhances patient benefits and extends overall survival (Spigel et al., 2022) in curative radiotherapy but also exerts synergistic therapeutic effects (Welsh et al., 2020) in patients with advanced disease.

However, the clinical efficacy of radiotherapy is often impeded by several challenges, with radioresistance being the primary concern. Radioresistance is a complicated biological process associated with an abnormal DNA damage response, apoptosis, autophagy, gene mutations, cell cycle checkpoints, and deregulated signaling pathways (Ni et al., 2017). Resistance to radiotherapy can be attributed to the intrinsic radioresistance of tumor cells within the hypoxic microenvironment or resistance acquired during fractionated radiotherapy (Toulany and Rodemann, 2015; Raja et al., 2014). This can lead to treatment failure and disease recurrence, posing a significant barrier to effective cancer management, making it a major obstacle for radiotherapy (Davidoff et al., 2011). Recent advances in research have provided deeper insights into the mechanisms underlying radioresistance, emphasizing the critical roles of both tumor cells and the tumor microenvironment (TME) (Horsman et al., 2012). Various factors such as DNA repair pathways (Rao et al., 2023a), replication stress (Zhang et al., 2022a), epigenetic regulation (Jie et al., 2021), cellular signaling networks, and the immune microenvironment contribute to the development of radioresistance (Sharma and Johnson, 2020). Consequently, further studies regarding these mechanisms is imperative to enhance therapeutic outcomes and improve the survival rates of patients with NSCLC.

Exosomes are small membrane-bound vesicles, approximately 30–150 nm in diameter, which are secreted by cells. These vesicles play crucial roles in intercellular communication by transferring various biomolecules, including proteins, lipids, RNA, and DNA (Kalluri and LeBleu, 2020). Exosomes were initially believed to function in cellular waste disposal. However, recent studies have shown that they perform significant regulatory functions in both physiological and pathological processes (Pegtel and Gould, 2019).

Exosomes exhibit significant heterogeneity, which is evident in their size, content, effects on recipient cells, and cellular origins. This diversity is primarily influenced by their biogenesis and the type of cells they originate from, including whether they are derived from cancer cells. These factors impart unique characteristics to exosomes, such as their propensity to target specific organs and incorporation by specific cell types (Kalluri and LeBleu, 2020; Vagner et al., 2019). Exosomes have garnered substantial attention in cancer research, particularly regarding their roles in tumorigenesis and cancer progression (Zhou et al., 2016). Tumor-derived exosomes (TDEs) can promote tumor growth, invasion, and metastasis and play a pivotal role in shaping the TME (Xie et al., 2019; Chen et al., 2021). Exosomes also modulate gene expression and behavior in recipient cells by transferring tumor-associated factors, such as miRNAs and proteins, thereby facilitating tumor progression (Guo et al., 2021).

Moreover, the relationship between exosomes and radiotherapy has become a focal point of research. Exosome release from tumor cells increases during radiotherapy, and they are implicated in the development of radioresistance (Shao et al., 2018). Further, radiotherapy-induced exosomes can deliver anti-apoptotic signals, enhance DNA repair, and modulate immune responses, thereby augmenting tumor cell radioresistance (Mutschelknaus et al., 2016; Jiang et al., 2020; Thomas et al., 2013; McLaughlin et al., 2020). Therefore, a thorough understanding of the mechanisms via which exosomes influence radiotherapy can offer new perspectives for overcoming radioresistance and enhancing the efficacy of radiotherapy.

Despite progress in research on exosome-mediated functions based on *in vitro* models, research on exosomes and radiotherapy in oncology still faces many challenges. Notably, there is a significant gap in literature involving comprehensive reviews that integrate the latest findings and provide a holistic understanding of exosome-induced radioresistance, particularly in the context of NSCLC. This review focuses on studies on NSCLC, reviewing the latest advancements in research on exosome-induced radioresistance. It also discusses how exosomes mediate radioresistance via the TME, advancements in mesenchymal stem cell (MSC)-derived exosome research, and the potential of exosomes and their transported miRNAs as biomarkers. This review highlights the importance of exosomes in cancer radioresistance and progression and provides a reference for the development of new therapeutic strategies.

## 2 Exosomes and radioresistance in NSCLC

**FIGURE 1:**
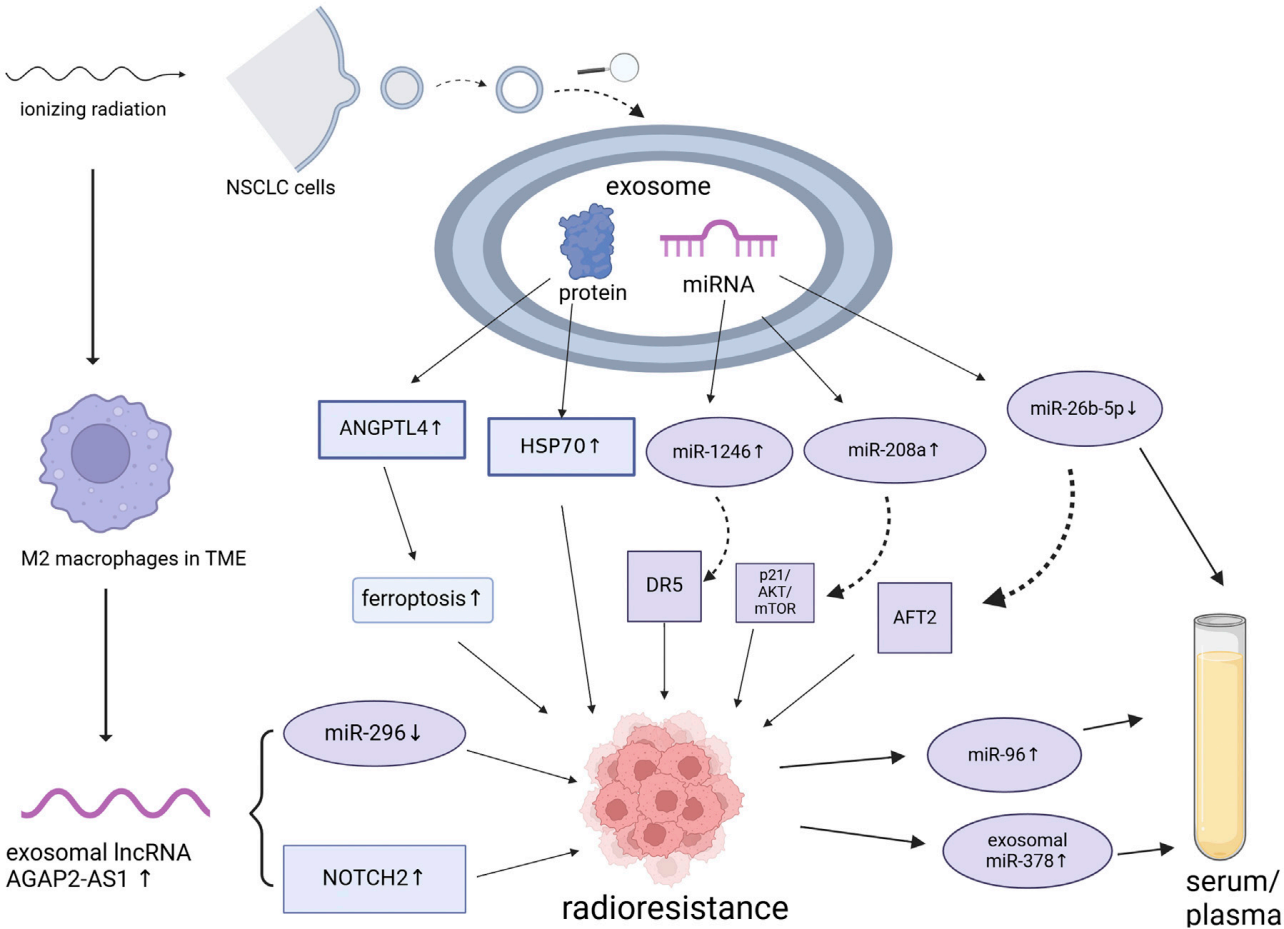
Presumptive mechanisms underlying the formation of ionizing radiation-induced exosomes, resulting in the radioresistance of non-small cell lung cancer. NSCLC, non-small cell lung cancer; miRNA, microRNA; TME, tumor microenvironment. Created using BioRender.

Despite continuous advancements in radiotherapy techniques, many patients with cancer, especially those with locally advanced cancers, still experience radiotherapy failure (radioresistance), which leads to local recurrence or distant metastasis. As discussed previously, radioresistance can emerge due to genetic or phenotypic alterations within the tumor or shielding against radiation by the tumor stroma and microenvironment. Accumulating evidence indicates that exosomes contribute significantly to the induction of radioresistance. Tang et al. (2016) observed a significant increase in serum miR-208a levels following radiotherapy in patients with lung cancer. This was found to enhance the proliferation and radioresistance of NSCLC cells via targeting of p21 and activation of the AKT/mTOR pathway, which promotes lung cancer cell proliferation and decreases cell apoptosis. Notably, exosomal miR-208a can be absorbed by other NSCLC cells, further enhancing their radioresistance. These findings underscore the potential of miR-208a to act as a therapeutic target and improve the efficacy of radiotherapy in patients with NSCLC. Moreover, Yuan et al. (2016) showed that the levels of the exosomal miR-1246 increases significantly in NSCLC cells and that it enhances radioresistance by targeting death receptor 5. These exosomal miRNAs not only facilitate intercellular communication but also directly affect tumor cell proliferation and survival by modulating key genes. Despite evidence from prior studies suggesting that exosomes play a pivotal role in enhancing cancer radioresistance, opinions on this matter remain divergent. Wang et al. (2019) recently reported that autocrine secretions enhance the radioresistance of the H460 NSCLC cell line in an exosome-independent manner, primarily by affecting DNA repair processes. Therefore, the role of exosomes in cancer radioresistance is complex and may be influenced by various factors, such as the tumor type, TME, experimental methods, or different treatment combinations. The presumptive mechanisms underlying the formation of ionizing radiation-induced exosomes, resulting in radioresistance of NSCLC is shown in Figure 1 and Table 1. However, this complexity must be explored in future studies.

**TABLE 1:** Roles of exosomal contents in modulating the radioresistance of non-small cell lung cancer (NSCLC).

Exosomal content	Source	Mechanism	Function	References
miR-208a	Cancer cells	Target p21 and AKT/mTOR pathway	Decrease cellular apoptosis and disturb the cell cycle to induce radioresistance	Tang et al. (2016)
miR-1246	Cancer cells	Directly target DR5	Promote cell proliferation and radioresistance	Yuan et al. (2016)
ANGPTL4	Cancer cells	Inhibit ferroptosis in exosomal ANGPTL4–GPX4-dependent manner	Confer radioresistance in hypoxic ells and transmit it to normoxic cells	Zhang et al. (2022b)
lncRNA AGAP2-AS1	M2-TAMs	Reduce miRNA-296 and elevate NOTCH2	Enhance radiotherapy immunity	Zhang et al. (2021)
miR-101-3p	BMSCs	Target EZH2 to activate the PI3K/AKT/mTOR pathway	Inhibit DNA damage repair and enhance radiosensitivity	Sun et al. (2024)
miR-378	Serum	Not yet clarified	Exhibit higher levels in patients with NSCLC and is associated with unfavorable prognosis	Zhang and Xu (2020)
miR-96	Plasma	Not yet clarified	Overexpressed in patients with NSCLC and correlated with vascular invasion and poor overall survival	Zheng et al. (2021)
miR-26b-5p	Cancer cells	Inhibit ATF2 expression	Promote DNA damage, apoptosis, and radiosensitivity	Han et al. (2020)
HSP70	Cancer cells	Not yet clarified	Prognostic and predictive relevance of pre- and post-treatment Hsp70 levels after RT	Ostheimer et al. (2017)

HSP70, heat shock protein 70; M2-TAMs, M2 tumor-associated macrophage; BMSCs, bone mesenchymal stem cells; DR5, death receptor 5; RT, radiotherapy.

## 3 Exosomes and the DNA damage response

Ionizing radiation is utilized in radiation therapy to damage cancer cells, generating intermediate ions and free radicals, particularly by targeting DNA, leading to single- or double-strand breaks. These breaks activate various signaling pathways that mediate the DNA damage response (DDR), a crucial determinant of the effectiveness of radiotherapy (Zhang et al., 2022a). The most relevant DDR processes involve double-strand break repair pathways, including homologous recombination and non-homologous end-joining (Li et al., 2001; Swift et al., 2023). Some studies have indicated an association between exosomes and the DDR. Jiang et al. (2020) reported that exosomal miR-194-5p from radiation-damaged pancreatic cancer cells enhances tumor cell regeneration. They also found that dying tumor cells release many exosomes early after radiotherapy, which further enhances the DDR and promotes the survival of tumor-regenerating cells. Mo et al. (2018) confirmed that exosomes from normal human bronchial epithelial BEP2D cells display increased expression of miRNA-1246 when induced by radiation. The authors also observed that the increase in miRNA-1246 expression enhanced both cell proliferation and colony formation. In contrast, Takahashi et al. (2017) observed that diminishing exosome secretion induces reactive oxygen species-dependent DDR in both senescent and non-senescent cells. This study revealed that exosome secretion inhibits anomalous activation of the DDR in pre-senescent cells, indicating that exosome secretion maintains cellular homeostasis by preventing erroneous activation of the DDR in certain types of normal human cells.

The relationship between exosomes and the DDR remains inconclusive, with current research indicating that the functions and contents of exosomes are complex and variable. Therefore, caution should be exercised when interpreting these findings. Further studies are required to elucidate these molecular mechanisms using a broader range of cell lines and animal models, along with standardized measurement techniques, which will clarify the specific effect of exosomes and their contents on the DDR.

## 4 Role of exosomes in the TME

The TME consists of immune cells, blood vessels, extracellular matrix, fibroblasts, lymphocytes, bone marrow-derived inflammatory cells, and signaling molecules surrounding the tumor cells. Moreover, continuous, dynamic, and reciprocal interactions between tumor cells and their microenvironment provide essential support for tumor cell survival. Tumor cell activities, such as proliferation, resistance to cell death, invasion, metastasis, angiogenesis, and immune evasion rely heavily on the TME (Arneth, 2019; Xiao and Yu, 2021). The key cell types in the TME include fibroblasts, endothelial cells, and infiltrating immune cells, which primarily communicate with tumor cells via exosome signaling. The nature of these interactions depends on the source and content of the exosome (Kohlhapp et al., 2015; Record et al., 2011). Hypoxia, high lactate levels, extracellular acidosis, and low nutrient availability are important TME markers. These conditions also stimulate tumor cells to release more exosomes than normal cells, inducing changes and amplification within the TME, which promotes tumor progression (Zhang and Grizzle, 2011; Thai et al., 2021). Hypoxia is a key factor involved in radiation resistance, as oxygen is required for generating the free radicals that are essential for ionizing radiation-mediated killing of tumor cells. Hypoxia also induces cellular transformations that prevent the harmful effects of radiotherapy (Horsman et al., 2012). Radio-biological hypoxia occurs when oxygen tension in the TME reduces below 0.13 kPa, interfering with radiation-induced cell death (Salem et al., 2018). Zhang et al. (2022b) showed that ANGPTL4, carried by exosomes derived from lung cancer cells under hypoxic conditions, mediates radioresistance in NSCLC by inhibiting ferroptosis and reducing lipid peroxidation. More importantly, they found that exosomes derived from hypoxic NSCLC cells could transmit radioresistance signals to the surrounding normoxic NSCLC cells in an exosomal ANGPTL4–GPX4-dependent manner. Thus, hypoxia-induced exosomal signaling plays a crucial role in regulating intercellular communication within the TME.

Cancer-associated fibroblasts (CAFs) are key components of the TME (Kim and Bae, 2016). Several theories regarding the origin of CAFs exist, including the differentiation of myofibroblasts into fibroblasts, transdifferentiation of epithelial and endothelial cells into fibroblasts via epithelial-mesenchymal transition, or the conversion of mesenchymal stem cells into fibroblasts (Xouri and Christian, 2010). Exosomes can transfer miR-9 to human mammary fibroblasts, transforming them into CAFs (Baroni et al., 2016). Tumor-derived exosomes have been shown to enhance the expression and signaling of transforming growth factor-beta (TGF-β) in mesenchymal cells (Baroni et al., 2016). For instance, exosomes from gastric, ovarian, and breast cancer can induce the conversion of mesenchymal stem cells into fibroblast-like cells, marked by increased levels of α-smooth muscle actin and phosphorylated Smad2 (Cho et al., 2012; Cho et al., 2011; Gu et al., 2012). Additionally, exosomes from gastric cancer cells can convert mesenchymal stem cells from umbilical cord blood into CAFs (Gu et al., 2012). These findings indicate that exosomes promote the acquisition of pro-tumorigenic characteristics by stromal cells within the TME.

TDEs play a crucial role in regulating the TME by mediating angiogenesis, immune evasion, and tumor-associated macrophage (TAM) polarization (Park et al., 2019; Tang et al., 2022). Understanding the mechanisms via which exosomes and their contents regulate the TME may help elucidate radiation resistance-associated pathways and guide the development of more effective exosome-based tumor radiotherapy strategies. Generally, rapid tumor growth induces hypoxia, which stimulates tumor cells to secrete more TDEs, further promoting angiogenesis and radiation resistance. Chen et al. (2022) demonstrated that exosomal miR-30b-5p from hypoxic pancreatic cancer cells promotes tumor angiogenesis by inhibiting the expression of gap junction alpha-1. Additionally, Chen et al. (2021) found that exosomes from esophageal squamous cell carcinoma under hypoxic conditions promote radiation resistance via miR-340-5p transfer.

TAMs are derived from monocytes in the bone marrow, which are a subset of TME cells that infiltrate most human tumors and differ from those in adjacent or normal tissues (Huang et al., 2019). Exosomes can stimulate macrophage polarization into M1 or M2 phenotypes, with the latter promoting angiogenesis, matrix remodeling, distant metastasis, and immunosuppression (Tian et al., 2019a; Baig et al., 2020). Exosomes transport functional miRNAs that regulate TAM infiltration and polarization. In addition, TDEs can directly or indirectly influence signaling pathways or gene expression to induce macrophage polarization. For example, exosomes from small cell lung cancer induce M2 polarization via the NLRP6/NF-κB pathway (Rao et al., 2022). Moreover, exosomes from gallbladder cancer cells containing high leptin levels promote M2 macrophage polarization via STAT3 signaling and enhance gallbladder cancer cell invasion and migration (Zhao et al., 2022). Wan et al. demonstrated that microparticles released from irradiated tumor cells elicit broad anti-tumor effects, primarily via ferroptosis, leading to immunogenic cell death. These molecules also facilitate M2 tumor-associated macrophage (M2-TAM) repolarization to M1-TAMs, thereby modulating the anti-tumor interactions between TAMs and tumor cells (Wan et al., 2020a). Subsequently, the same research group engineered irradiated tumor cell-released microparticles harboring the SARS-CoV-2 spike protein and TGFBR2 as vaccines. The aim of this approach was to stimulate the innate immune response and suppress the immunosuppressive TME, ultimately enhancing T cell activation and infiltration (Sun et al., 2023). Zhang et al. (2021) reported that the exosomal long non-coding RNA, AGAP2-AS1, derived from M2 macrophages, enhances the immunoeffectiveness of radiotherapy by downregulating miR-296 expression and upregulating NOTCH2 levels. In particular, AGAP2-AS1 expression was found to be significantly upregulated in radioresistant NSCLC cells, whereas miR-296 expression was significantly downregulated and NOTCH2 expression was significantly increased. Moreover, inhibition of AGAP2-AS1 expression significantly reduced the survival and proliferation of radioresistant NSCLC cells.

The TME comprises functional immunomodulators and immune cells, mainly controlled by peripheral blood and lymphatic vessels, which facilitate information exchange between primary or metastatic tumor sites and host immune organs (Hiam-Galvez et al., 2021). TDEs can induce immunosuppressive phenotypes in immune cells, promoting their infiltration into tumor tissues and leading to immunosuppression. This interference affects antigen presentation by dendritic cells, activation and proliferation of T and B cells, and cytotoxicity of natural killer cells (Tian et al., 2019b; Tesi, 2019). For example, exosomes from EGFR-19del Lewis lung cancer cells transfer active EGFR-19del to dendritic cells, inducing an unresponsive state that inhibits anti-tumor immunity (Yu et al., 2020). Furthermore, exosomes containing interleukin-32γ from multiple myeloma enhance macrophage PD-L1 expression via protease 3, promoting glycolysis-dependent immune evasion (Liu et al., 2022). In summary, TDEs play a significant role in TME formation and development, mediating the behavior and progression of tumor cells. Ultimately, further elucidation of the specific mechanisms via which exosomes and their contents influence various types of tumors will provide new directions for cancer diagnosis and treatment.

## 5 MSC-derived exosomes and radioresistance

MSCs comprise a group of cells with self-renewal and multipotent differentiation capabilities (Pittenger et al., 1999). These cells can be derived from various human tissues and components, including the bone marrow, adipose tissue, dental pulp, umbilical cord, menstrual blood, and amniotic fluid. Owing to their multipotency, MSCs can differentiate into multiple cell types, including osteocytes, chondrocytes, adipocytes, and myocytes, making them valuable tools for regenerative medicine (Lootens et al., 2024).

Extensive research has confirmed that MSCs are essential components of the TME and are associated with cancer progression (Whiteside, 2018). As key players in TME reprogramming, MSCs play a crucial role in cell-to-cell communication by transmitting important cellular information that influences the growth, differentiation, and functions of neighboring cells (Wang et al., 2022). These cells can also modulate immune cell activities, assist in the transition of macrophages to the M2 type, inhibit neutrophil functions, and direct the differentiation and proliferation of B and T lymphocytes (Shan et al., 2024). Furthermore, MSCs control tumor cell growth by secreting various substances, such as cytokines, chemokines, and growth factors (Wang et al., 2022; Vizoso et al., 2017). Notably, several studies have indicated that MSC-derived exosomes play a crucial role in regulating resistance to cancer therapy by mediating the exchange of substances and signals between cancer cells and other stromal cells (Weng et al., 2021). Wan et al. (2020b) discovered that miR-34c, delivered by MSC-derived exosomes, significantly inhibits the proliferation, migration, and radiotherapy resistance of nasopharyngeal carcinoma cells. Furthermore, Li et al. (2020) found that the expression of miR-101-3p is reduced in irradiated NSCLC tissues and cells, confirming that miR-101-3p enhances NSCLC radiosensitivity in animal experiments. Sun et al. (2024) further demonstrated that exosomal miR-101-3p from bone marrow MSCs enhances NSCLC radiosensitivity by regulating EZH2 and promoting DDR and tumor cell autophagy. We believe that detailed investigation regarding MSC-derived exosomes holds great promise. These tiny vesicles released by MSCs have intriguing properties that could be pivotal for enhancing the effectiveness of cancer radiotherapy. By broadening our understanding regarding their mechanisms and functions, we can design novel approaches for improving therapeutic outcomes. Therefore, research in this field should be boosted to utilize the full spectrum of benefits that MSC-derived exosomes offer in the fight against cancer.

## 6 Role of exosomes as biomarkers after tumor radiotherapy

Exosomes, known for their unique properties, can act as biomarkers for tumor diagnosis and prognosis when present after tumor radiotherapy (Gao et al., 2021). They can also be used in liquid biopsies to predict the efficacy of radiotherapy and optimize treatment plans. Moreover, changes in miRNA levels within the serum/plasma exosomes of patients with tumors after radiotherapy can indicate the effectiveness of the treatment (Fan et al., 2022). Alterations in specific miRNA levels can further be used as indicators to predict the therapeutic outcomes of tumor radiotherapy (Pu et al., 2020). Dinh et al. (2016) demonstrated that among the 752 exosomal miRNAs identified in patients with locally advanced NSCLC, the expression of miR29a-3p and miR150-5p decreased with increasing radiation doses, indicating their potential to act as early biomarkers that correlated with the delivered radiotherapy dose. Additional studies (Zhang and Xu, 2020) have shown that exosomal miR-378 is significantly upregulated in patients with NSCLC and is associated with lymph node metastasis and an advanced TNM stage. Post-radiotherapy, serum exosomal miR-378 levels reduce significantly, indicating its potential as a marker of radiotherapy response. Survival analyses further revealed that patients with high serum exosomal miR-378 levels have poor overall survival. These findings further support the crucial role of exosomal miRNAs in modulating the response to radiotherapy. Zheng et al. (2021) identified circulating exosomal miR-96 as a novel biomarker for radioresistant NSCLC. Their study showed that exosomal miR-96 levels are significantly higher in patients with NSCLC than in controls and are even higher in patients with radioresistant NSCLC. Han et al. (2020) found that exosomal miR-26b-5p, enhances the radiosensitivity of lung adenocarcinoma cells after downregulating ATF2. They also found that compared to those in patients with high miR-26b-5p expression, patients with lung adenocarcinoma and low serum levels of miR-26b-5p have significantly shorter survival periods. This suggests that miR-26b-5p may act as a potential non-invasive biomarker for predicting response to radiotherapy and prognosis of lung adenocarcinoma. In addition, Ostheimer et al. (2017) demonstrated that dynamic changes in serum heat shock protein 70 (HSP70) levels could be used to predict the clinical response of patients with NSCLC to radiotherapy. In particular, serum HSP70 levels before treatment correlated significantly with the expression of hypoxia-related marker, osteopontin (OPN), and high OPN levels indicated poor overall survival. HSP70 levels decreased significantly after radiotherapy; however, patients with higher post-treatment HSP70 levels exhibit better radiotherapy responses and longer survival. These findings suggest that monitoring the dynamic changes in HSP70 levels before and after radiotherapy can provide additional prognostic information, aiding in the rapid adjustment of treatment plans.

The possibility of using exosomes in liquid biopsies following tumor radiotherapy is being actively investigated. Several studies have focused on the cargo carried by exosomes and their effect on tumor invasiveness after radiotherapy, as well as their use in evaluating radiotherapy efficacy and prognosis and optimizing treatment plans. Considering that many potential biomarkers have not yet been identified and validated, further research on exosome-based biomarkers is urgently required. These future studies should consider their clinical utility and potential value in clinical practice.

## 7 Perspectives and conclusion

Radiation therapy is an indispensable component of the comprehensive treatment regimen of lung cancer and plays a significant role in both early-stage unresectable and advanced-stage lung cancer treatment. In particular, in the era of immunotherapy, the role of radiation therapy has become more important than before. The results of the I-SABR study showed that for early-stage NSCLC without lymph node and distant metastases, the 4-year event-free survival rate can reach 77% in patients treated with a combination of a PD-1 inhibitor and SABR, considerably improving the survival of patients with early-stage lung cancer who are unable to undergo surgical treatment (Chang et al., 2023). The standard treatment model for stage III lung cancer was revised based on the observations of the PACIFIC study, with maintenance immunotherapy after concurrent chemoradiotherapy or sequential radiotherapy increasing the 3-year survival rate to approximately 60% (Antonia et al., 2017; Girard et al., 2023). However, the failure of the PACIFIC-2 study also discouraged the use of immunotherapy combined with radiotherapy, suggesting that further categorization of patients with lung cancer benefiting from radiation therapy is still required.

Basic research has indicated that radiation damage has an immune-activating effect; however, immune cells are more sensitive to radiation damage than cancer cells. Numerous studies have focused on optimization of radiation therapy targets, innovating radiotherapy techniques, and reducing the irradiation range and dose to protect anti-tumor immune cells (Khandekar and Keane, 2021). However, radiation resistance remains an issue. First, accurately identifying patients with lung cancer who are resistant to radiation is crucial as it determines whether a higher localized dosage or individualized radiosensitizers should be administered to enhance treatment efficacy. Second, predicting radiosensitive patients to minimize their dosage helps protect immune cell functions, strengthen immune responses, and simultaneously reduce the side effects of radiation (Rao et al., 2023b). Therefore, assessing radiosensitivity in patients with lung cancer, understanding the mechanisms of radiation resistance, and developing effective sensitization strategies remain challenges in the field of radiation oncology. Although most current research is at the preliminary stage of elucidating these mechanisms, we believe that exosomes will be highly valuable targets for overcoming radioresistance in cancer therapy.

In oncology, exosomes are considered potential biopredictors because they can be released into the extracellular space and detected in various types of body fluids. The levels of exosomes can be compared between different groups in a simple, feasible, and cost-effective manner (Shin et al., 2023). Additionally, exosomes are considered personalized therapeutic carriers because they can transport bioactive molecules, such as proteins, RNA, and DNA, delivering these molecules to other cells, thereby influencing tumor behavior and the microenvironment. Researchers are investigating whether exosomes can be used to deliver therapeutic molecules, such as anticancer drugs, gene-editing tools, or immune-modulating molecules, to enhance the immune response of tumors or directly inhibit tumor growth (Liang et al., 2021; Tian et al., 2023). Moreover, targeted delivery to particular cell types can be achieved by engineering exosomes with specific surface markers, thereby improving the precision and efficacy of treatment (Sun et al., 2023; Xu et al., 2020a). Research regarding the role of and differences in exosomes between various subtypes of NSCLC is underway. In patients harboring *EGFR* mutations, the same mutations have been identified in plasma exosomes, supporting their potential as a diagnostic tool (Rao et al., 2023c; Liu et al., 2024). Additionally, exosomal PD-L1 has been associated with the effectiveness of immunotherapy in patients with lung cancer (Shimada et al., 2021; Kim et al., 2019). These findings suggest that exosomes can partially reflect the genetic background of the tumor, along with associated malignant behavior, treatment sensitivity, and tumor burden. However, despite the immense potential of exosomes in cancer therapy, challenges remain, including the large-scale production of homogeneous exosomes, ensuring their safety, and optimizing their therapeutic payload. Consequently, scientists are actively conducting experiments and clinical studies to overcome these obstacles and spearhead exosome-based therapeutic strategies for clinical applications.

Information regarding the changes in exosomal content during radiation response may be used for diagnosis and development of therapy. With progress in our understanding of exosomal miRNAs, the combination of miRNA-based therapies and radiotherapy might offer a promising personalized treatment strategy. However, several challenges must be overcome before the current exosome–miRNA research can be translated into clinical practice. First, elucidation of the radiobiological molecular mechanisms and targets of miRNAs is fundamental for designing miRNA-based therapies in the future. Tumor progression during radiotherapy is dynamic and associated with varying levels of miRNA expression. Thus, rigorous clinical studies should carefully consider the differences in the TME and continuously monitor miRNA levels throughout the course of radiotherapy. Furthermore, utilization of noninvasive liquid biopsies to harness the potential of exosomes is currently challenging. Despite significant efforts to standardize these techniques, consensus regarding the optimal method for isolating and quantifying exosomes is lacking. To date, the clinical applicability of exosomal miRNAs in radiotherapy has been limited because their potential functions have not been adequately studied in patient samples. Further prospective investigations involving patients undergoing radiotherapy are crucial to validate this clinical utility. Finally, a single miRNA cannot be used to consistently predict clinical outcomes. A comprehensive analysis of miRNAs may help improve their performance, and the prediction accuracy of a combination of different biomarkers may be better than that of individual markers. Moreover, real-time monitoring of these markers can increase their predictive value. Therefore, further research is required to develop new circulating markers and investigate the predictive capabilities of previously identified peripheral blood markers based on the tumor tissue.

In addition to acting as biomarkers, exosomes constitute a research hotspot in the fields of cancer development, progression, migration, invasion, metastasis, and therapy. An increasing number of studies has confirmed that exosomes promote tumor development, suggesting that inhibition of their biogenesis, secretion, and uptake elicits anti-tumor behavior (Chai et al., 2024; Dai et al., 2020). Moreover, owing to their structural characteristics, exosomes can protect the transported molecules from degradation during delivery, enabling them to cross various barriers, including the blood–brain barrier, and can spread throughout the body to reach distant tissues. Recently, their potential for drug delivery has been recognized (Kosaka et al., 2010; Andaloussi et al., 2013; Ha et al., 2016). Kim et al. (2018) reported macrophage-derived exosomes loaded with paclitaxel combined with an aminoethyl carbamate-polyethylene glycol carrier fragment. These exosomes targeted the sigma receptors that are overexpressed in lung cancer cells and their systemic administration led to drug accumulation in cancer cells, thereby improving therapeutic efficacy. Moreover, Bai et al. (2020) constructed a novel targeted tLyp-1 exosome via genetic engineering, which showed high efficiency in transfecting lung cancer and cancer stem cells. Compared to traditional gene- or cell-based therapies, drug delivery mediated by exosomes offers a compelling alternative because of their reduced immunogenicity, smaller size, and ability to traverse biological barriers (Lu et al., 2023a; Lu et al., 2023b).

In 2013, the Nobel Prize in Physiology or Medicine was awarded to James E. Rothman, Randy W. Schekman, and Thomas C. Südhof for their groundbreaking discoveries of the machinery regulating vesicle traffic. Since then, the therapeutic potential of these phospholipid bilayer-enclosed extracellular vesicles in various diseases have been investigated. For instance, drug-loaded vesicles have shown promising results in treating malignant pleural effusion by offering targeted, low-toxicity, and highly effective anti-tumor immune activation (Xu et al., 2020b; Guo et al., 2019). Lin and colleagues have demonstrated that extracellular vesicles produced by radiation therapy can deliver immune activation signals to T cells. Using vesicle proteomics, they identified a new tumor-associated antigen, CDCP1, specific peptide fragments of which can elicit an immune response that effectively inhibits tumor growth (Lin et al., 2020). However, the use of exosomes as therapeutic carriers is associated with challenges. One major issue is the difficulty in scaling up exosome isolation and purification while retaining only the tumor-treating components and removing those that promote tumor growth. Additionally, as exosomes possess biological immunogenicity, preventing them from triggering an immune response in the host remains a significant hurdle. Furthermore, exosomes are prone to degradation during storage and transport, which may impact their therapeutic efficacy. Improving the biological stability of exosomes is one of the focus areas of current research. In the future, synthetic vesicles with enhanced stability and safety, engineered alongside tumor-targeting drugs, proteins, peptides, or even miRNAs, may be created to achieve more precise therapeutic delivery.

At present, one of the main obstacles preventing exosome therapy from entering clinical practice is the low yield and efficiency of exosome production. For example, less than 1 μg of exosomal protein can be obtained from 1 mL of culture medium in a laboratory setting (Gurunathan et al., 2019). Additionally, exosomes exhibit heterogeneity in size, content, surface markers, and origin, which complicates their isolation. The currently available exosome isolation and purification techniques are based on size, surface charge, and immunoaffinity (Kimiz-Gebologlu and Oncel, 2022). However, no method is perfect, and each has its advantages and disadvantages. For example, ultracentrifugation is considered the gold standard for exosome extraction. While it requires minimal reagents and expertise, its time-consuming nature, high cost, low efficiency, and the co-isolation of lipoproteins limit its use for large-scale applications (Yang et al., 2019). Immunoaffinity chromatography is a separation technique based on the specific binding of antibodies to ligands. This method is fast and highly pure exosomes can be obtained with high specificity and yield. However, the antigens or proteins used for conjugation should be expressed on the exosome surface (Doyle and Wang, 2019). Particle size-based separation techniques, such as ultrafiltration and size-exclusion chromatography, are rapid and suitable for large-scale applications. However, challenges such as pore clogging, exosome loss, and low purity make these methods less ideal for widespread use (Kalluri and LeBleu, 2020). Although no single technique is perfect, combining the aforementioned methods with other methods, such as precipitation-based and microfluidic-based techniques, may ensure that the various requirements of exosome isolation and purification are met.

Currently, the clinical trials database (https://www.clinicaltrials.gov) lists over 30 entries focused primarily on the applications of exosomes in lung cancer, including diagnosis, treatment, and efficacy analysis. In particular, exosome-related clinical research has been extended to various types of cancer, covering areas such as the identification of biomarkers for diagnosis and prognosis, therapeutic interventions, drug delivery mechanisms, and the development of cancer vaccines. However, the clinical application of exosomes in NSCLC radiotherapy is still in its early stages and is mostly limited to *in vitro* studies. Researchers face many challenges that must be addressed in the future, including gaining a deeper understanding of the biological characteristics of exosomes and their contents, standardizing exosome isolation and identification methods, and exploring how exosomes affect the microenvironment and cellular signaling pathways during radiotherapy. With continuous technological advancements, we believe that once the limitations related to scaling-up of production, adherence to good manufacturing practices, and compliance with regulatory frameworks are overcome, exosome-related therapies will soon be incorporated into clinical practice. This will bring innovative treatments to the bedside and benefit more patients with cancer (Wang et al., 2020).

## Abbreviations

NSCLC, non-small cell lung cancer; TME, tumor microenvironment; TDE, Tumor-derived exosome; DDR, DNA damage response; CAFs, cancer-associated fibroblasts; TAM, tumor-associated macrophage; MSC, mesenchymal stem cell.
